# Identifying chronic thromboembolic pulmonary hypertension through the French national hospital discharge database

**DOI:** 10.1371/journal.pone.0214649

**Published:** 2019-04-18

**Authors:** V. Cottin, D. Avot, L. Lévy-Bachelot, C. A. Baxter, D. R. Ramey, L. Catella, S. Bénard, O. Sitbon, S. Teal

**Affiliations:** 1 National Reference Centre for rare pulmonary diseases, Competence centre for pulmonary arterial hypertension, Louis Pradel hospital, Claude Bernard University Lyon 1, UMR 754, Lyon, France; 2 MSD France, Courbevoie, France; 3 MSD Ltd, Hoddesdon, United Kingdom; 4 Merck & Co., Inc., Kenilworth, NJ, United States of America; 5 stève consultants, Oullins, France; 6 Université Paris-Sud, CHU de Bicêtre, Le Kremlin-Bicêtre, France; 7 Bayer AG, Berlin, Germany; Vanderbilt University Medical Center, UNITED STATES

## Abstract

Chronic thromboembolic pulmonary hypertension (CTEPH), a rare pulmonary vascular disease, is often misdiagnosed due to nonspecific symptoms. The objective of the study was to develop, refine and validate a case ascertainment algorithm to identify CTEPH patients within the French exhaustive hospital discharge database (PMSI), and to use it to estimate the annual number of hospitalized patients with CTEPH in France in 2015, as a proxy for disease prevalence. As ICD-10 coding specifically for CTEPH was not available at the time of the study, a case ascertainment algorithm was developed in close collaboration with an expert committee, using a two-step process (refinement and validation), based on matched data from PMSI and hospital medical records from 2 centres. The best-performing algorithm (specificity 95%, sensitivity 70%) consisted of ≥1 pulmonary hypertension (PH) diagnosis during 2015 and any of the following criteria over 2009–2015: (i) CTEPH interventional procedure, (ii) admission for PH and pulmonary embolism (PE), (iii) PE followed by hospitalization in competence centre then in reference centre, (iv) history of PE and right heart catheterization. Patients with conditions suggestive of pulmonary arterial hypertension were excluded. A total of 3,138 patients hospitalized for CTEPH was estimated for 2015 (47 cases/million, range 43 to 50 cases/million). Assuming that patients are hospitalized at least once a year, the present study provides an estimate of the minimal prevalence of CTEPH and confirms the heavy burden of this disease.

## Introduction

Pulmonary hypertension (PH) is defined by a mean pulmonary arterial pressure ≥25 mm Hg at right heart catheterization (RHC). Although this definition is the same for all PH, there are differences between subtypes in the underlying mechanisms, and most importantly, for prognosis and therapeutic implications [1]. PH is clinically categorized into five WHO groups according to similar clinical presentation, pathological findings, haemodynamic characteristics and optimal treatment strategy. Chronic thromboembolic pulmonary hypertension (CTEPH, PH Group 4) is characterized by the presence of organized fibrous tissue (chronic thromboembolic material) occluding varying degrees of the pulmonary arterial tree. Survival rates at 1 year, 2 years, and 3 years are approximately 93%, 91%, and 89%, respectively [2]. Patients should be evaluated for suitability of pulmonary endarterectomy (PEA), which can be curative [3–5], although some patients may still experience symptoms after the procedure. Patients who are considered inoperable, or with persistent/recurrent PH post-PEA are candidates for balloon pulmonary angioplasty (BPA) or targeted medical therapy, according to CTEPH treatment guidelines [1].

Since CTEPH is rare and may be underdiagnosed, the overall prevalence of CTEPH is difficult to evaluate [1]. It has been estimated in a recent meta-analysis of cohort studies that CTEPH may occur in up to 3.2% of survivors of acute pulmonary embolism (PE) [6], and in 4.8% of PE survivors according to a French cohort study (95% CI [2.3; 9.6]) [7]. Moreover, approximately 25% of CTEPH cases may occur in patients without a known history of PE [8]. To date, only two registries have published epidemiological data [9,10]: Spain (PH registry) and Portugal (CTEPH/pulmonary arterial hypertension (PAH) registry) reported an estimated annual incidence of 0.9 and 1.1 CTEPH cases per million inhabitants, respectively, and a prevalence of 3.2 per million inhabitants for the Spanish registry. However, these figures must be interpreted carefully due to the small number of CTEPH patients included in the registries (n = 162 and 33, respectively), and should be considered as minimum estimates of incidence. The prevalence of CTEPH is estimated at 30 per million inhabitants in Europe by Orphanet (based on non-specified review of European data) [11], which would correspond to approximately 2,000 cases in France. A recent epidemiological study provided a different estimate for the prevalence for the year 2015 in France of 45 per million, corresponding to approximately 3,000 patients [12].

In France, CTEPH management is organized around a national reference centre, and affiliated regional centres in university hospitals (known as competence centres). All patients with CTEPH in France are likely to be hospitalized periodically since the disease is typically managed on an inpatient basis and the procedure of RHC, necessary to confirm CTEPH diagnosis and to follow the course of the disease, is also generally performed during a hospitalization. As no specific International Classification of Diseases, Tenth edition (ICD-10) code was available for CTEPH at the time of the study, those patients are typically classified as "secondary PH" (I27.2), along with patients from WHO PH group 1 (PAH associated with other conditions), 2 (PH with left heart disease), 3 (PH due to lung diseases and/or hypoxia) and 5 (PH with unclear and/or multifactorial mechanisms) [1]. As no straightforward identification based on ICD-10 codes exists, ascertainment of CTEPH cases needs to be based on relevant combinations of diagnoses and medical procedures, excluding conditions suggestive of other WHO PH groups.

The objective of this study was to develop, refine and validate a case ascertainment algorithm to identify patients with CTEPH in the PMSI database (Part I), and to use this algorithm to estimate the number of CTEPH patients hospitalized in France in 2015 (Part II). The French exhaustive national hospital discharge database (PMSI) covers all French public hospitals and private clinics involved in medicine, surgery and obstetrics, and was therefore considered as particularly relevant for estimating the number of CTEPH patients hospitalized annually, as a proxy of CTEPH prevalence.

## Materials and methods

### Study overview

A retrospective study was developed, based on matched data from PMSI and hospital medical records from 2 centres (Kremlin Bicêtre [KB] in Paris, the reference centre in France and Louis Pradel [LP] in Lyon, a competence centre). A two-step process was conducted: a preliminary algorithm was refined in the training database (KB hospital), followed by a validation of the final algorithm in LP hospital. The final algorithm was then used to estimate the annual number of CTEPH patients hospitalized in France in 2015, after applying a correction based on the final algorithm performance.

A scientific committee comprising two pulmonologists (VC, OS) was established to provide clinical and practical expertise regarding methodological choices adopted during the refinement and validation of the algorithm.

### Data sources

#### PMSI database

The French exhaustive national hospital discharge database PMSI covers all stays in French public and private hospitals. The PMSI also includes home care (*hospitalisation à domicile*—HAD), psychiatric stays (*Recueil d’Information Médicalisée en Psychiatrie—*RIM-P), and post-acute care and rehabilitation (*Soins de suite et de réadaptation*—SSR), but for the study purposes, only stays from medicine, surgery, obstetrics and odontology (*Médecine*, *chirurgie*, *obstétrique et odontologie*—MCO) were used [13]. From 2004, French hospitals have adopted a prospective payment system, for which the PMSI database has become the basis of hospital funding by third-party payers, ensuring exhaustive completion. A standard discharge summary report is generated for each hospital stay and includes information on the patient’s characteristics (e.g. sex, age, residence code), diagnoses, and examinations carried out during hospitalization. Diagnoses are coded using ICD-10, either as main diagnosis (one per stay), related diagnosis (one per stay) or significant associated diagnoses (as many as necessary). Medical procedures are coded using the French *Classification Commune des Actes Médicaux* (CCAM). For the purpose of this study, case ascertainment algorithms were designed on the basis of ICD-10 diagnosis codes, on medical procedures codes, and on unique hospital number (indicating the type of centre: reference, competence or other).

#### Ethics statement

Agreement to extract and use the PMSI data was obtained from the French data protection authority, the National Commission on Informatics and Liberty (CNIL, authorization number 1559750). Data included in the PMSI are anonymized and comply with the French legislation about confidentiality and data protection. Informed consent from the participants is not required. Data on confirmed cases of CTEPH for the first centre (KB hospital, authorization number 1396054) and those extracted from the French PH registry (authorization number 842063) are also in accordance with the requirements of the CNIL. Agreement for linking both data sources with the PMSI was approved by the CNIL (authorization DR-2017-069).

#### Confirmed CTEPH patients based on medical charts

Confirmed CTEPH cases were obtained from the medical charts of two centres which both maintain a prospective, exhaustive registry. Data on confirmed cases of CTEPH for the KB hospital were available from a prospective database set up for observational research (*tableau de bord du centre de référence hypertension artérielle pulmonaire et requêtes patients*). Confirmed cases from the second centre (LP) were extracted from the French PH registry, which was opened in 2002, and planned to enrol all consecutive patients aged ≥18 years with PH [14–16].

#### Data linkage

PMSI data were matched to KB and LP hospitals’ clinical databases in order to identify confirmed CTEPH patients. In order to identify the common patients in each clinical database and PMSI, the following variables were used: age, sex, ZIP code, admission and discharge dates, type of hospitalization (inpatient or day), medical procedures performed (including RHC). A deterministic approach [17] was then used for linking both datasets (exact matching required between variables chosen for linkage). Patients with PMSI data without corresponding CTEPH records from the respective centres were considered as not having CTEPH.

For the refinement step, the training population comprised patients hospitalized at least once for PH (ICD-10 code I27) in the KB hospital in 2015 and living in Ile-de-France area (to exclude patients referred from other regions in France to this Reference centre). For the validation step, the population comprised patients hospitalized at least once for PH (ICD-10 code I27) in the LP hospital in 2015.

### Part I. Development, refinement and validation of case ascertainment algorithm to identify patients with CTEPH in the PMSI database

#### Development of the Preliminary Case Ascertainment Algorithm

In the absence of specific ICD-10 code for CTEPH, ascertainment of CTEPH cases needs to be based on PH code (I27) and relevant combinations of diagnoses and medical procedures intended to be specific of CTEPH management, excluding conditions suggestive of other WHO PH-groups. Expert interview (pulmonologist at the Louis Pradel hospital, Reference Center for Rare Pulmonary Diseases and Rhône-Alpes Competence Center for Pulmonary Arterial Hypertension) and literature review were conducted in order to identify the typical care pathway for managing CTEPH patients. Two extensive literature reviews were performed on the 20^th^ of July 2015 through Medline with a limit of time up to 10 years. For the first review on patient care management, a total of 57 publications were identified and 3 publications were selected. In addition, 3 publications were identified through grey literature. For the second on epidemiological data, 179 publications were identified and 27 publications were selected. In addition, 4 epidemiological publications of interest were identified through grey literature.

From experts’ knowledge, after CTEPH diagnosis, patients are referred to a competence centre and, next, they are referred to the reference centre to assess their eligibility for PEA. Since 2014, patients not eligible for PEA or with persistent/recurrent PH after PEA may now undergo a BPA. Patients who have undergone interventional procedures, *i*.*e*. PEA or BPA (after 2014), could be identified in the PMSI using specific CCAM codes for the procedures (S1 Table).

As PEA is a specific procedure to treat CTEPH patients, a review of PMSI hospitalization records was first performed in order to identify specific care patterns (diagnoses, medical procedures, type of centre) that could be included in the case ascertainment algorithm. To note, some medications could be prescribed for CTEPH patients, but could not be used for identification in this study as they are not encoded in the PMSI [18].

Algorithms used in previous database studies [19–22] were also analysed. In the CPRD study by Martinez et al. [19], a cohort of patients with a history of venous thromboembolism was first selected. CTEPH cases were then identified by a discharge diagnosis for PH in hospital records or keywords from free text notes within medical records. There were three claims database studies conducted in the US [20–22] which used a similar algorithm to identify CTEPH patients: at least 2 claims for PH, with the first claim occurring at least 6 months after a claim for PE, and at least 1 claim for RHC or echocardiogram before the second PH claim. This review of previous algorithms showed the interest of first selecting PH patients, then adding additional selection criteria based on PE or RHC for instance.

Finally, the exploration of coding patterns in PMSI for confirmed CTEPH patients, in addition to expert interviews and review of algorithms used in previous database studies [19–22], allowed us to build a preliminary CTEPH case ascertainment algorithm, which was reviewed and revised based on the input and experience of the scientific committee. The following structure for the algorithms was retained, with a view to applying it to a population with at least one hospital stay with a diagnosis of PH. The first criterion (called “entry criterion”) comprised interventional procedures: PEA or BPA. In contrast with PEA, BPA is not specific for CTEPH management and has been developed for other disorders (e.g., congenital pulmonary stenosis). A concomitant diagnosis (i.e. within the same stay) for PH is then required along with the procedure code for BPA, and the procedure had to be performed in selected hospitals, authorized at the time of the study to perform this procedure for CTEPH patients. The second entry criterion included hospital stay for PH and PE (ICD-10 code I26) in time-relevant order to select PH after PE, suggesting CTEPH. The third entry criterion looked for multiple PE events, also suggestive of CTEPH when associated with PH diagnosis. The fourth entry criterion aimed to capture CTEPH patients managed according to a specific CTEPH care pathway, including diagnostic suspicion, diagnostic confirmation in a competence centre, and then referral to the reference centre for assessing their eligibility for surgery. The fifth entry criterion consisted of history of PE and one or more RHC (codes in S1 Table). RHC is a procedure generally performed during a hospitalization, required for CTEPH diagnosis and also used during disease course, expected to be less specific but previously used for identifying CTEPH in other database studies [19–22].

All these criteria were independent, and patients needed to meet only one to be included. The preliminary algorithm was the following: ≥1 hospital stay with a diagnosis of PH over one year (2015) and any of the following criteria over 2009–2015: (i) history of PEA or BPA associated with PH diagnosis, (ii) hospital stay for PH and PE, (iii) history of ≥ 3 PE, (iv) CTEPH care pathway: hospital stay for PE, then for PH in competence centre, then for PH in a reference centre, (v) history of PE and 1 RHC.

#### Algorithm refinement and validation

Sensitivity, specificity, positive and negative predictive values (PPV and NPV) of different case ascertainment algorithms designed from the procedure described above were determined, using medical charts from the KB training database as the gold standard. After a refinement step performed on the KB training database, the algorithm exhibiting the highest specificity (true positives) was chosen (final algorithm), and then validated against the validation set (LP database).

True positives (TP) were those correctly identified as CTEPH through PMSI data, while true negatives (TN) were those correctly identified as not having CTEPH through PMSI data. False positives (FP) were those identified as having CTEPH through PMSI data, but not retrieved in the CTEPH reference standard from the KB and LP hospitals, and false negatives (FN) were those not identified as having CTEPH through PMSI data but retrieved in the CTEPH reference standard. Sensitivity, specificity, PPV and NPV were computed accordingly, along with 95% confidence intervals (CI) computed using normal approximation or binomial 'exact' method. Statistical analyses were performed using SAS 9.4 (SAS Institute Inc, Cary, NC). The method of validation was reported in accordance with the modified Standards for Reporting of Diagnostic Accuracy criteria, STARD 2015 [23] as detailed in S2 File and S1 Fig.

### Part II. Application of the algorithm for identifying hospitalized patients with CTEPH

The annual number of hospitalized patients with CTEPH was obtained after applying the final case ascertainment algorithm to all PMSI hospitalizations in France for 2015. In order to adjust this estimate based on the final algorithm performance, a corrected number of hospitalized patients with CTEPH was computed by (i) removing the estimated number of false positives and (ii) adding the estimated number of false negatives obtained through the validity assessment of the final algorithm [24,25]. In addition, low and high estimates of the number of hospitalized CTEPH patients were computed to provide a range taking into account the statistical variability associated with the performance indicators. The low CI estimate used the lower limit of the 95% CI of the performance indicators (Se, PPV) for computing the number of false positives to subtract and the number of false negatives to add, whereas the high CI estimate used the upper limit of the 95% CI of the performance indicators (Se, PPV) for computing the number of false positives to subtract and the number of false negatives to add (S1 File).

## Results

### Part I. Case ascertainment algorithm refinement and validation

**Fig 1:**
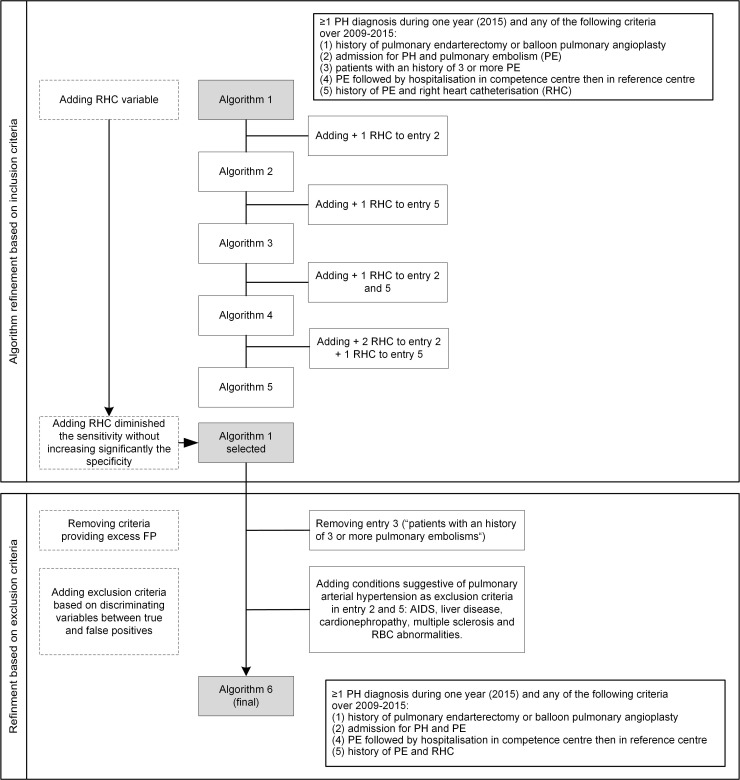
Flow diagram of the refinement step.

Records from KB hospital were obtained for 170 newly diagnosed CTEPH patients in 2015, with 159 patients (93.5%) successfully retrieved in the PMSI for the year 2015. For the LP hospital, 56 out of the 57 CTEPH patients (98.2%) were successfully retrieved. In parallel, data from 1,022 patients hospitalized at least once for PH (ICD-10 code I27) in the KB hospital in 2015 and living in Ile-de-France area were extracted to build the training set (1,160 for the validation set in LP hospital). In total, 6 algorithms were developed and tested before selecting the final one. The stepwise process for selecting the algorithm is presented in the flow diagram Fig 1.

The best performing algorithm (specificity 95%, sensitivity 70%) with a negative predictive value of 99% and a positive predictive value of 41% consisted of ≥1 PH diagnosis for one year (2015) and any of the following criteria over 2009–2015: (i) history of PEA or BPA associated with PH diagnosis, (ii) hospital stay for PH and PE, excluding conditions suggestive of PAH: acquired immune deficiency syndrome (AIDS), liver disease, cardionephropathy, multiple sclerosis or red blood cell count (RBC) abnormalities, (iii) CTEPH care pathway: hospital stay for PE, then for PH in competence centres, then for PH in a reference centre, (iv) history of PE and 1 RHC, excluding conditions suggestive of PAH (see ii). Performance of case ascertainment algorithms is detailed in Table 1.

**Table 1:** Performance of tested case ascertainment algorithms on training and validation sets (medical charts records as the reference standard).

	True-positive/ false-positive	True-negative/ false-negative	Sensitivity (%) (95% CI)	Specificity (%) (95% CI)	PPV (%) (95% CI)	NPV (%) (95% CI)
**Algorithm definition**						
1st algorithm (*)	132/123	17/750	88.6 (83.5–93.7)	85.9 (83.6–88.2)	51.8 (45.6–57.9)	97.8 (96.7–98.8)
2nd algorithm (* +1 RHC in entry 2)	121/102	28/771	81.2 (74.9–87.5)	88.3 (86.2–90.4)	54.3 (47.7–60.8)	96.5 (95.2–97.8)
3rd algorithm (* +1 RHC in entry 5)	128/120	21/753	85.9 (80.3–91.4)	86.3 (83.4–88.5)	51.6 (45.4–57.8)	97.3 (96.1–98.4)
4th algorithm (* +1 RHC in entry 2, +1 RHC in entry 5)	117/99	32/774	78.5 (71.9–85.1)	88.7 (86.6–90.7)	54.2 (47.5–60.8)	96.0 (94.7–97.4)
5th algorithm (* + 2 RHC in entry 2 + 1 RHC in entry 5)	101/78	48/795	67.8 (60.2–75.2)	91.1 (89.1–93.0)	56.4 (49.2–63.7)	94.3 (92.7–95.9)
6th algorithm (* entry 3 removed) on training set	129/75	20/798	86.6 (81.1–92.1)	91.4 (89.6–93.3)	63.2 (56.7–69.9)	97.6 (96.5–98.1)
**6th algorithm (final)** **on validation set**	**38/55**	**16/1,051**	**70.4 (58.2–82.5)**	**95.0 (93.7–96.3)**	**40.9 (30.9–50.8)**	**98.5 (97.8–99.2)**

CI, Confidence Interval; NPV, Negative Predictive Value; PPV, Positive Predictive Value; RHC, right heart catheterization

* First/preliminary algorithm: ≥1 PH diagnosis during one year (2015) and any of the following criteria over 2009–2015: (i) history of PEA (medical procedures-CCAM code) or BPA (ICD-10 code of PH and a CCAM code of BPA), (ii) hospital stay for PH and PE, (iii) history of ≥3 PE, (iv) CTEPH care pathway: hospital stay for PE, then for PH in competence centre, then for PH in a reference centre, (v) history of PE and 1 RHC.

### Part II. Number and characteristics of hospitalized patients with CTEPH

**Fig 2:**
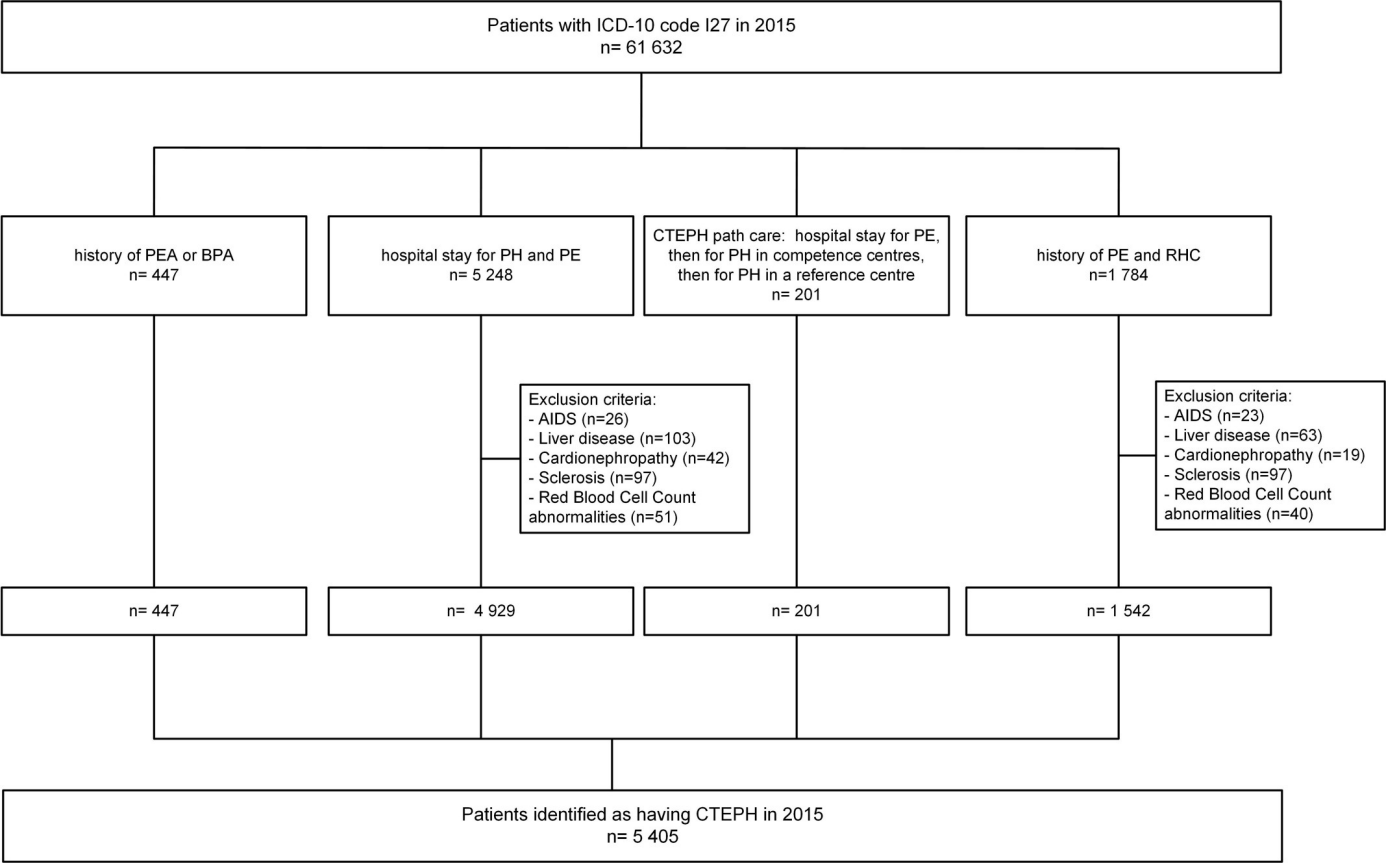
Selection using the final case ascertainment algorithm.

Application of the final algorithm to the 61,632 patients hospitalized at least once for PH in 2015 in the national PMSI database, identified 5,405 patients as having CTEPH (Fig 2).

After adjustment of the initial estimate based on the algorithm performance, the estimated annual number of hospitalized patients with CTEPH in 2015 was 3,138 (47 cases per million), as detailed in Table 2.

**Table 2:** Annual number of hospitalized patients with CTEPH in France before and after adjustment according to the algorithm performance.

	Patients	Low CI estimate^a^	High CI estimate^b^
**Annual number of hospitalized CTEPH patients**	5,405	—	—
Number of false positives subtracted	3,197	3,737	2,657
Number of false negatives added	930	1,199	581
**Corrected number of hospitalized CTEPH patients**	3,138	2,867	3,330
**Cases / million**^**c**^	47	43	50

CI, Confidence Interval; CTEPH, Chronic thromboembolic pulmonary hypertension

^a^Low CI estimate: use the lower limit of the 95% confidence interval (CI) of the performance indicators (Se, PPV) for computing the number of false positives to subtract and the number of false negatives to add.

^b^High CI estimate: use the upper limit of the 95% confidence interval of the performance indicators (Se, PPV) for computing the number of false positives to subtract and the number of false negatives to add.

c with a denominator of 66.381 million for the French population in 2015

## Discussion

Considering the scarcity and uncertainty of data published in the literature about the prevalence of CTEPH, the present study provides evidence on the epidemiology of CTEPH based on a validated case ascertainment algorithm built from matched data from PMSI and hospital medical records. Even though the preferred algorithm did not have optimal sensitivity and PPV, an approach taking into account the performance of the algorithm led to a corrected prevalence estimate of 47 cases per million.

### External validity

Our finding can be compared with previous epidemiological studies on CTEPH. The prevalence of CTEPH was estimated at 30 per million inhabitants in Europe by Orphanet (based on non-specified review of European data) [11], which would correspond to ~2,000 cases in France. According to experts’ opinion, the incidence of CTEPH would be around 300 to 400 new cases per year in France [26]. Only a few other studies have reported epidemiological data on patients with CTEPH. Registries from Spain [9] and Portugal [10] reported a minimum incidence of 0.9 and 1.1 per million inhabitants respectively, and an estimated prevalence of 3.2 per million inhabitants for the Spanish CTEPH registry. These data must be interpreted carefully due to the small number of patients with CTEPH within these registries (n = 162 and 33, respectively). Some studies based on insurance claims have also provided epidemiological estimates. A US study, which was reviewed for building our algorithm, reported prevalence estimates of 63 and 1,007 per million inhabitants, for patients under the age of 65 and older than 65, respectively [21].

Another recent epidemiological analysis in the US, Europe and Japan, provided an estimate for the prevalence for the year 2015 in France [12]. This study used PMSI data for estimating PE rates, but the final epidemiological estimates were based on several consecutive assumptions from the literature. Based on PE rates from PMSI in 2011, a projection using PE survival rates (based on US data) was performed, then an estimate of CTEPH rates in PE patients was applied (using data from the literature [27–34]). In addition, an estimate of CTEPH without documented prior PE [8] was added to approach the incidence of CTEPH. For 2015, this study provided an estimate of 45 cases per million, corresponding to ~3,000 patients, after applying an incidence to prevalence coefficient of 4.6 to the 650 newly diagnosed (incident) patients estimated, and using a French population figure of 66.381 million [12]. This study and ours therefore reach comparable results using different methodologies, which should be considered as an external validation of our study. The resulting estimate of the number of hospitalized patients with CTEPH in France, while subject to some limitations, should be considered as the most accurate and robust estimate that could be obtained through the data sources and methods available at the time of the study. The estimate is based on national data and a case ascertainment algorithm with known performance characteristics.

### Strengths and limitations

Some limitations must be discussed. Due to the limited sample size of the validation set, the confidence intervals for sensitivity and PPV were quite wide. The associated statistical uncertainty was taken into account in the sensitivity analyses using the lower and upper limits of the 95% confidence intervals. In spite of its very high specificity, the algorithm produced a considerable number of false negatives, with a moderate sensitivity of 70.4%. False negatives are patients with PH but no history of PE, no PEA or BPA, and no hospital stay in the reference centre recorded in PMSI. Even if it is unlikely that incident hospital stays for PE or PEA/BPA are not coded in the PMSI, the reference standard databases are assumed to be exhaustive in such case. To set up the case ascertainment algorithm, we used all specific available variables in the PMSI. Of note, it was not possible to evaluate the use of PH-specific medications in the studied population in the PMSI database. Only therapies on the “costly drugs list” are recorded, and this does not include all available therapies for PH [18].The frequency of CTEPH in the general population is expected to be lower than in the population used for refinement and validation, due to the use of a convenience sample comprising the population with at least one PH diagnosis. Since PPV and NPV are dependent on the prevalence of the disease in the population of interest, they would be expected to be modified when applied to a non-selected population [35]. However, this is a limitation commonly observed in validation studies [36], which should not affect further use of the algorithm when it is mainly focused on intrinsic parameters (specificity, sensitivity) that are not affected by disease prevalence. In addition, the reference standard for CTEPH status was based on a review of the medical charts from two expert referral centres with high volume of clinical activity related to PH patients management. As a result, the characteristics of patients (particularly age) and the habits of ICD coding might slightly differ between hospitals, potentially leading to variability. Management of patients with CTEPH, however, is expected to be relatively homogeneous thanks to published guidelines [1].

## Conclusions

The present study provides an estimate of over 3,000 hospitalized patients with CTEPH in France in 2015 among ~67 million inhabitants and confirms the heavy burden of this disease. This result may represent a solid estimate of the current prevalence of CTEPH in France, assuming that patients are hospitalized at least once a year.

## Supporting information

S1 Fig STARD flow diagram showing the numbers receiving the index test (algorithm) and reference standard (CTEPH hospital charts) (validation of the final algorithm against LP data). (TIF)

S1 File Method used for computing the number of false positives to subtract and the number of false negatives to add for the correction based on algorithm performance. (DOCX)

S2 File STARD checklist. (DOCX)

S1 Table CCAM codes used for ascertaining interventional procedures. (DOCX)
