# Supplementary figures and images for: Identifying chronic thromboembolic pulmonary hypertension through the French national hospital discharge database
Source: PLoS One. 2019 Apr 18;14(4):e0214649. doi: 10.1371/journal.pone.0214649 (PMC6472741; doi:10.1371/journal.pone.0214649)

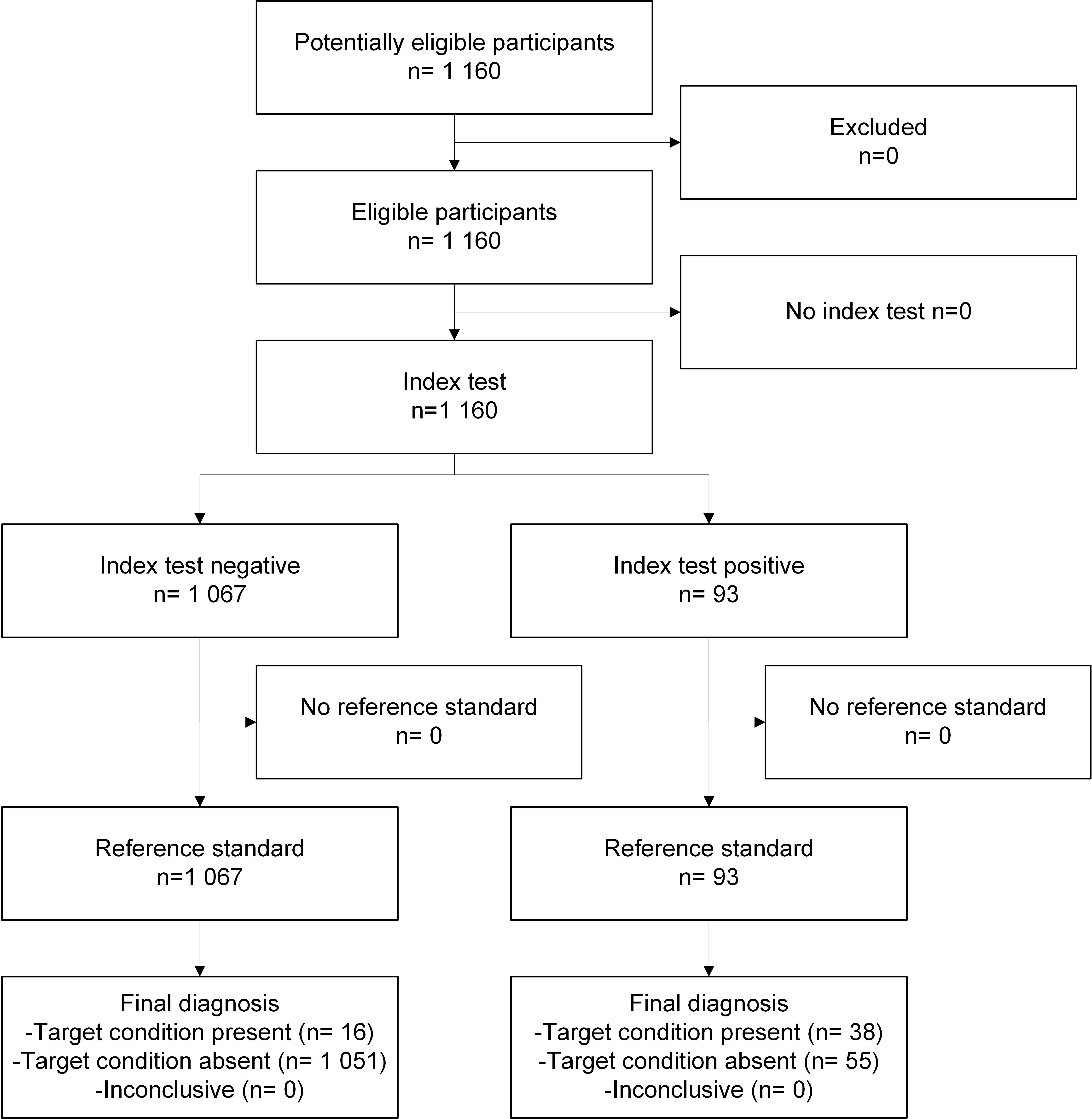

Supplement: S1 Fig — (TIF) [file pone.0214649.s001.tif]
