# Supplementary material for: Identifying chronic thromboembolic pulmonary hypertension through the French national hospital discharge database
Source: PLoS One. 2019 Apr 18;14(4):e0214649. doi: 10.1371/journal.pone.0214649 (PMC6472741; doi:10.1371/journal.pone.0214649)
Supplement: S1 File — (DOCX) [file pone.0214649.s002.docx]

# Supporting information

**S1 File. Method used for** **computing the number of false positives to subtract and the number of false negatives to add for the correction based on algorithm performance**

**Estimated number of false positives to subtract**= Total Positive^a^ - (Total Positive*PPV/100) *(1)*

**Estimated number of false negatives to add** = Total Positive^a^ – False positive^b^*(1 - Se /Se) *(2)*

CI, Confidence Interval; Se, sensitivity; NPV, Negative Predictive Value; PPV, Positive Predictive Value

^a^ Total positive: Number identified as having CTEPH after applying the case ascertainment algorithm on PMSI data

^b^ Number of false positives estimated through the formula *(1)*

Main estimate: use the rounded value (3 decimal place) of the performance indicators (PPV, Se) for computing the number of false positives to subtract and the number of false negatives to add

Low CI estimate: use the lower limit of the 95% CI of the performance indicators (PPV, Se) for computing the number of false positives to subtract and the number of false negatives to add

High CI estimate: use the upper limit of the 95% CI of the performance indicators (PPV, Se) for computing the number of false positives to subtract and the number of false negatives to add
