# Supplementary material for: Identifying chronic thromboembolic pulmonary hypertension through the French national hospital discharge database
Source: PLoS One. 2019 Apr 18;14(4):e0214649. doi: 10.1371/journal.pone.0214649 (PMC6472741; doi:10.1371/journal.pone.0214649)
Supplement: S1 Table — (DOCX) [file pone.0214649.s004.docx]

# Supporting information

**S1 Table. CCAM codes used for ascertaining interventional procedures**

| **Interventional procedures** | **CCAM code** | **Original label (French)** |
| --- | --- | --- |
| Pulmonary endarterectomy (PEA) | DFFA003 | Thromboendartériectomie du tronc et/ou des branches de l'artère pulmonaire, par thoracotomie avec CEC |
| Balloon pulmonary angioplasty (BPA) | DFAF001 | Dilatation intraluminale de plusieurs branches de l'artère pulmonaire avec pose d'endoprothèse, par voie veineuse transcutanée |
|  | DFAF002 | Dilatation intraluminale d'une branche de l'artère pulmonaire sans pose d'endoprothèse, par voie veineuse transcutanée |
|  | DFAF003 | Dilatation intraluminale d'une branche de l'artère pulmonaire avec pose d'endoprothèse, par voie veineuse transcutanée |
|  | DFAF004 | Dilatation intraluminale de plusieurs branches de l'artère pulmonaire sans pose d'endoprothèse, par voie veineuse transcutanée |
| Right heart catheterization (RHC) | EQQF003 | Mesure et enregistrement des pressions intravasculaires pulmonaires et systémiques et des différences artérioveineuses des contenus artériels en oxygène, à l'état basal et après administration d'agent pharmacologique vasodilatateur, par voie vasculaire transcutanée |
|  | EQQF006 | Mesure et enregistrement des pressions du coeur droit et de l'artère pulmonaire, sans injection de produit de contraste, par voie veineuse transcutanée |
